# Pharyngeal electrical stimulation for postextubation dysphagia after stroke: a randomized trial on hospitalization costs from a health insurance perspective

**DOI:** 10.1038/s41598-026-43591-9

**Published:** 2026-03-09

**Authors:** Bendix Labeit, Anne Jung, Jonas von Itter, Inga Claus, Sigrid Ahring, Tobias Warnecke, Paul Muhle, Almut Kremer, Sven G. Meuth, Rainer Dziewas, Sonja Suntrup-Krueger

**Affiliations:** 1 Department of Neurology, Medical Faculty, University Hospital Düsseldorf, Moorenstraße 5, 40225 Düsseldorf, Germany; 2 Department of Neurology, University Hospital Muenster, Albert-Schweitzer- Campus 1, A1, 48149 Muenster, Germany; 3 Department of Neurology and Neurorehabilitation, Klinikum Osnabrueck, Am Finkenhügel 1, 49076 Osnabrueck, Germany; 4 DRG Research Group, University Hospital Muenster, Albert-Schweitzer- Campus 1, A1, 48149 Muenster, Germany

**Keywords:** Post-stroke-dysphagia, Stroke, Pharyngeal electrical stimulation (PES), Healthcare costs, Diseases, Health care, Medical research, Neurology

## Abstract

Dysphagia is a frequent and serious complication after stroke, associated with pneumonia, malnutrition, prolonged intensive care unit stay, and higher healthcare costs. Pharyngeal electrical stimulation (PES) is a novel therapy that may support recovery from postextubation dysphagia. This study assessed the cost-effectiveness of PES in stroke patients from a health insurance perspective. We performed a secondary analysis of a randomized controlled trial including 60 stroke patients with postextubation dysphagia, randomized to PES (*n* = 30) or sham stimulation (*n* = 30). Sham treatment involved identical device placement without stimulation. Acute hospital costs were estimated per patient using the 2025 German Diagnosis-Related Groups (DRG) reimbursement system. Mean costs were compared between groups with a one-sided t-test, and distributions were explored with boxplots. Mean DRG costs per patient were €22,392.89 (SD = €14,980.84) in the sham group and €18,127.20 (SD = €7,828.65) in the PES group. The difference was not statistically significant (*p* = 0.087). However, three outliers with costs >€50,593 occurred in the sham group, compared with none in the PES group (maximum €35,257.49). Although the difference in mean hospitalization costs between groups was not statistically significant, extreme high-cost outliers occurred only in the sham group in this pilot sample. This observation is exploratory and hypothesis-generating and requires confirmation in larger, adequately powered health-economic studies before firm conclusions regarding cost-effectiveness can be drawn.

## Introduction

 Dysphagia is a common complication of acute stroke, affecting up to 75% of patients when assessed using instrumental gold-standard methods^1^. Its prevalence is particularly high among intubated patients, who often experience more severe strokes. Moreover, intubation itself can contribute to the development of peripheral postextubation dysphagia^2^. Conversely, dysphagia may also lead to (re)intubation and prolonged intensive care unit (ICU) stays due to its association with respiratory complications such as aspiration pneumonia^3^. Additional adverse outcomes include malnutrition, the need for nasogastric or percutaneous endoscopic gastrostomy (PEG) feeding, extended hospitalizations, poor functional recovery, and increased mortality^4^.

Importantly, respiratory complications after stroke and extubation are not determined solely by the presence of aspirated material but by the balance between aspirated load and the effectiveness of bronchial clearance mechanisms^4^. After prolonged intubation, mucociliary clearance may be substantially impaired due to airway instrumentation and cuff-related effects, rendering effective cough one of the most important remaining protective mechanisms. Consequently, pneumonia risk in this population depends not only on swallowing safety but also on cough strength, secretion management, and overall airway protection. In clinical practice, respiratory therapy and mechanical cough assistance are established strategies to improve secretion clearance and reduce pulmonary complications after extubation^5,6^.

Instrumental assessment is considered the gold standard for diagnosing dysphagia^4^. It is essential for detecting silent aspiration, which occurs without observable protective reflexes such as coughing, and for accurately grading dysphagia severity and the dysphagia phenotype to guide targeted interventions^4,7^. Flexible Endoscopic Evaluation of Swallowing (FEES) and Videofluoroscopic Swallow Study (VFSS) are both well-established and diagnostically equivalent methods for assessing clinically relevant dysphagia pathologies^8^. FEES is particularly well suited for bedside and intensive care settings^9,10^. The Fiberoptic Endoscopic Dysphagia Severity Scale (FEDSS), a validated six-point ordinal scale, enables structured assessment and provides clear management recommendations for patients with acute stroke^11^.

Current widely adopted treatments for dysphagia primarily rely on compensatory strategies that aim to reduce complications rather than restore swallowing function^12^. These strategies include optimized oral hygiene, dietary texture modifications, and nutritional support^12,13^. However, because swallowing is centrally controlled by a distributed network involving cortical, subcortical, and brainstem structures, neurostimulation techniques may facilitate recovery by promoting neural plasticity^4,12^. The bilateral cortical representation of swallowing further increases the potential for functional rehabilitation following stroke^4,12^.

Pharyngeal Electrical Stimulation (PES) is a neurostimulation therapy that involves transnasal insertion of a catheter to deliver electrical stimulation to the pharyngeal mucosa. Treatment typically consists of 10-minute sessions administered over three consecutive days, targeting afferent sensory pathways. Proof-of-concept studies have demonstrated that PES induces reorganization within the cortical swallowing network^14–16^. Clinically, phase II and III trials have shown its efficacy in improving swallowing function and facilitating decannulation in severely dysphagic, tracheostomized stroke patients^17–19^. However, in less severely affected stroke patients, its therapeutic effect remains controversial^12,13,20–22^.

In a recent randomized, sham-controlled pilot trial, we investigated early administration of PES in acute stroke patients with severe postextubation dysphagia^23,24^. The intervention was associated with a nonsignificant tendency to reduced reintubation rates (*p* = 0.067), faster recovery of swallowing function, improved airway protection, and shorter stays in both the ICU and the hospital. Given these clinical benefits, PES may also contribute to lower acute hospitalization costs, indicating potential cost-effectiveness. This secondary analysis evaluates hospitalization costs from a health insurance perspective by comparing PES-treated patients with sham controls from the original trial.

## Methods

### Study design

The present analysis is based on the full randomized cohort of the underlying randomized controlled trial (ClinicalTrials.gov NCT02470078)^23^, which was conducted between December 2015 and April 2018 at the certified stroke unit and neurological ICU of the University Hospital Münster, Germany. To facilitate replication, a concise summary of the trial population and patient flow is provided here. Consecutive adult patients with acute ischemic or hemorrhagic stroke within 14 days of onset who had been mechanically ventilated and had just undergone their first extubation attempt were screened immediately post-extubation. Severe postextubation dysphagia was identified using FEES and defined as FEDSS > 4, which served as the primary inclusion criterion.

Major exclusion criteria followed the parent trial protocol and included: (i) pre-existing swallowing impairment unrelated to stroke, (ii) comorbidities likely to independently cause dysphagia, (iii) implanted electronic devices, and (iv) extubation performed in a palliative setting. Written informed consent was obtained from patients or their legal representatives prior to enrollment. Randomization was performed in a 1:1 ratio to PES or sham stimulation within 4 h after extubation, and treatment was initiated within 2 h thereafter. PES was delivered using the Phagenyx^®^ catheter and stimulation system (Phagenesis Ltd., Manchester, United Kingdom), a nasogastric catheter-based device with integrated ring electrodes connected to a dedicated stimulation base unit, as described in the original trial protocol. Allocation was computer-generated and masked to outcome assessors and clinical caregivers, while all patients received standard stroke and dysphagia care according to national guidelines^9^.

In total, 78 patients were assessed for eligibility. Fifteen patients did not meet the inclusion criterion of severe postextubation dysphagia (FEDSS ≤ 4), two had an implanted cardiac pacemaker, and one declined participation; therefore, 60 patients were randomized. All randomized patients were included in the intention-to-treat population. In the PES group (*n* = 30), four patients did not receive the allocated intervention as per protocol (*n* = 2 no intervention; *n* = 2 one intervention session). In the sham group (*n* = 30), seven patients did not receive the allocated intervention as per protocol (*n* = 1 no intervention; *n* = 4 one intervention session; *n* = 2 two intervention sessions). As reported in the parent trial, the primary reason for incomplete intervention delivery was clinical deterioration or re-intubation during the 3-day intervention period. No patient was lost to follow-up.

All 60 randomized patients from the original trial were included in the present secondary cost analysis; no additional exclusion criteria were applied. Thus, the cohort analyzed here corresponds exactly to the intention-to-treat population of the parent randomized trial. Further methodological details are reported in the primary clinical publication^23^.

### Determination of expenditures by health insurers

The reimbursement amount that the hospital could bill health insurers according to the DRG-system (DRG revenue) was determined for each patient according to the 2025 billing guidelines^25^. In Germany, inpatient treatments are reimbursed through the Diagnosis Related Groups (DRG) system, a fixed rate, performance based payment model. Each hospital case is classified into a DRG based primarily on the principal diagnosis that prompted admission. Additional factors, including surgical and procedural codes (OPS), duration of mechanical ventilation, and secondary diagnoses, also influence DRG assignment.

To account for clinical complexity, the DRG system incorporates the Patient Clinical Complexity Level (PCCL), which quantifies the overall burden of comorbidities and complications. The PCCL is calculated using a standardized formula that aggregates the severity and relevance of secondary diagnoses. The total hospital length of stay also affects reimbursement. If a patient’s stay falls below or exceeds the predefined lower or upper length of stay thresholds for the assigned DRG, surcharges or deductions may apply.

DRG cost rates are determined by the German Institute for Hospital Remuneration (Institut für das Entgeltsystem im Krankenhaus, InEK), based on average treatment costs collected from designated reference hospitals. Comprehensive national guidelines regulate how hospital revenue is allocated per patient within this framework^25^.

For this analysis, we identified the DRGs assigned to each patient along with the corresponding total DRG revenues that the hospital could bill health insurers in 2025. These revenues include all reimbursements from statutory health insurance, except for nursing care payments (Pflegeentgelt) and specific supplementary reimbursements (Zusatzentgelt, Neue Untersuchungs- und Behandlungsmethoden). Only direct costs associated with acute inpatient care at University Hospital Muenster were considered. Costs related to rehabilitation, outpatient care, or subsequent hospital readmissions were not included.

### Statistics

All data handling and visualization were performed in Python (version 3.11) using pandas 2.2.3 for data management, SciPy 1.14.1 for statistical analyses, and matplotlib 3.7.5 together with seaborn 0.11.2 for figure generation and visualization. The objective was to compare direct treatment costs between the intervention group (“PES group”) and the control group (“sham group”). Descriptive statistics for the acute hospitalization costs (mean, median, standard deviation) were calculated for each group. Given the a priori directional hypothesis that the intervention would reduce costs, we conducted a one-sided Welch’s t-test to compare the difference in mean costs between groups. Cost distributions with determination of high-cost outliers were further visualized using boxplots. For each group, the first (Q1) and third quartile (Q3) were calculated and the interquartile range (IQR) was defined as Q3 − Q1. High-cost outliers were defined as cases exceeding Q3 + 1.5×IQR. Values outside these thresholds are displayed as individual data points in the box-plot.

To further explore potential drivers of exceptionally high hospitalization costs, an exploratory outlier analysis was conducted. Patients classified as high-cost outliers were descriptively compared with the remainder of the cohort. For these patients, ventilation duration (hours), total hospital length of stay (days), pneumonia, and comorbidity level were compared with the corresponding values in the non-outlier cohort. No formal hypothesis testing was performed for this analysis, which was considered exploratory and hypothesis-generating.

## Use of artificial intelligence

ChatGPT-4 and ChatGPT 5.2 (OpenAI) was used to assist with language editing and the drafting of illustrative figures. All content was reviewed and approved by the authors.

## Results

### Study cohort baseline characteristics

Baseline demographic, stroke-related, and swallowing parameters were comparable between the PES and sham groups at the time of study inclusion (Table 1, based on the parent randomized controlled trial^23^. Although the NIHSS score at admission was slightly higher in the PES group, no relevant differences were present at study inclusion, and stroke type, lesion location, etiology, acute treatment procedures, and ventilation duration prior to enrollment were similar between groups.

**Table 1:** Baseline characteristics of the study cohort: Values are mean ± standard deviation unless indicated otherwise.

Characteristic	PES group (*n* = 30)	Sham group (*n* = 30)	*p*-value
Age (years, mean ± SD)	69.5 ± 15.7	64.6 ± 12.7	0.185
Female, n (%)	19 (55)	13 (43)	0.121
NIHSS upon admission (pts)	16.4 ± 3.6	14.3 ± 4.1	0.041
NIHSS at study inclusion (pts)	13.7 ± 2.9	12.5 ± 3.0	0.090
Time from admission to study inclusion (days, mean ± SD)	3.3 ± 2.5	2.5 ± 2.6	0.066
Duration of mechanical ventilation (h, mean ± SD)	83.3 ± 95.3	64.7 ± 61.3	0.264
Type of stroke			
Ischemic stroke, n (%)	27 (90)	29 (97)	0.301
Hemorrhagic stroke, n (%)	3 (10)	1 (3)	
Site of stroke			
Infratentorial, n (%)	6 (20)	2 (7)	0.129
Supratentorial, n (%)	24 (80)	28 (93)	
Right / Left supratentorial, n	13 / 11	20 / 8	0.198
Stroke etiology			
Large-artery atherosclerosis, n (%)	9 (33)	5 (17)	0.165
Cardioembolism, n (%)	15 (56)	16 (55)	0.977
Small-vessel occlusion, n (%)	0 (0)	0 (0)	n.a.
Other determined etiology, n (%)	1 (4)	4 (14)	0.186
Unknown etiology, n (%)	2 (7)	4 (13)	0.440
Acute stroke treatment			
Intravenous thrombolysis, n (%)	16 (59)	11 (38)	0.110
Mechanical recanalization, n (%)	24 (89)	26 (90)	0.926
Decompressive craniectomy / hematoma evacuation, n (%)	5 (17)	4 (13)	0.718

NIHSS = National Institutes of Health Stroke Scale.

A complete baseline dataset was originally reported in the parent randomized controlled trial^23^.

### DRG-codes in the cohort

**Fig. 1:**
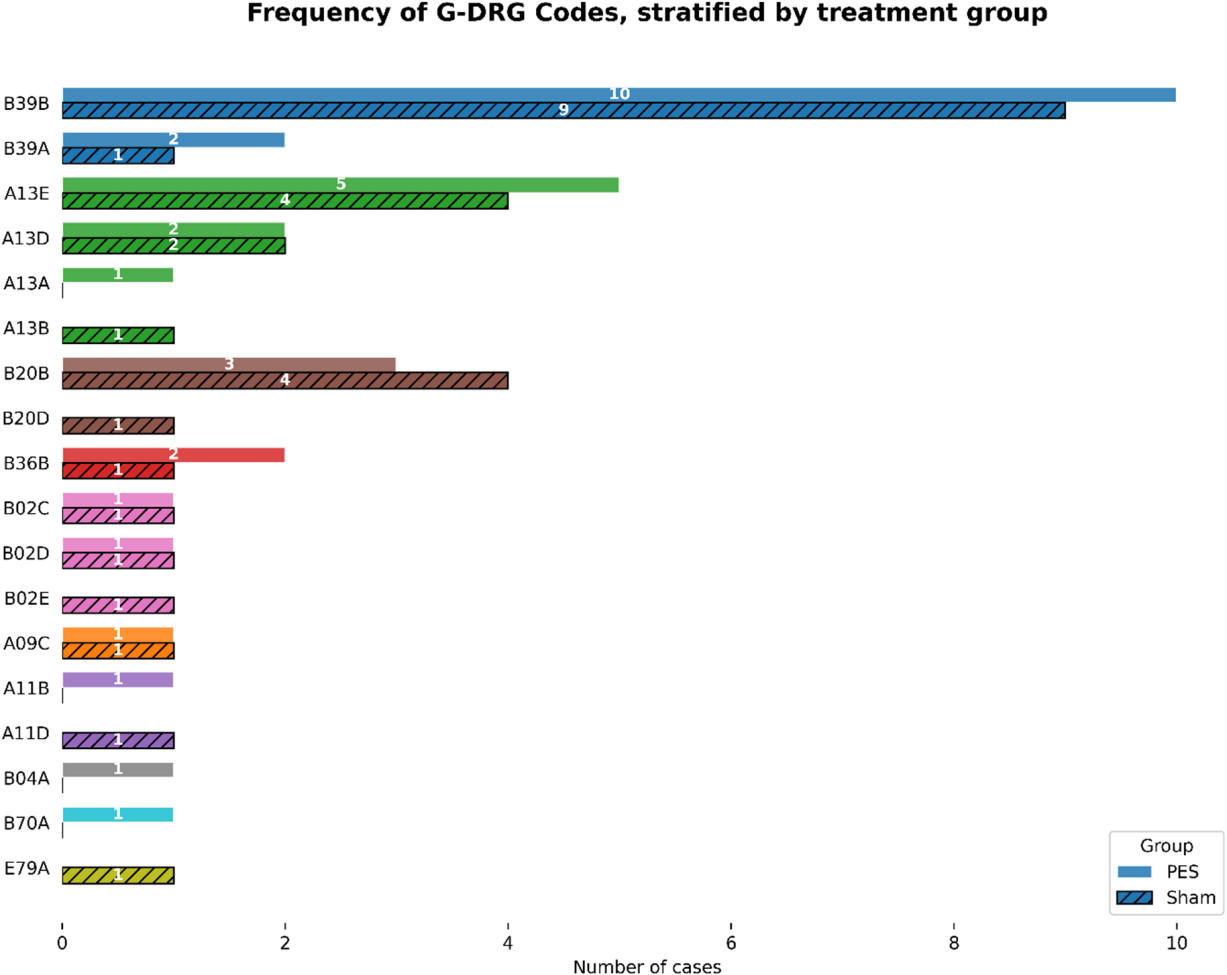
Frequency of German DRG codes stratified by treatment group (PES vs. sham). B39 (blue): Complex neurological treatment of acute stroke with specific OR procedure; A13 (green): Ventilation > 95 hours, without complex intensive care treatment > 1764 / 1656 / 1656 cost points; B20 (brown): Craniotomy or major spinal surgery; B36 (red): Intensive care complex treatment > 1176 / 1104 / 1104 effort points or > 588 / 552 / 552 effort points with specific OR procedure for diseases and disorders of the nervous system or specific highly complex implants; B02 (pink): Complex craniotomy or spinal surgery; A09 (orange): Ventilation > 499 hours or > 249 hours with complex intensive care treatment > 2352 / 1932 / 2208 effort points; A11 (orange): Ventilation > 249 hours or > 95 hours with complex intensive care treatment > 1764 / 1656 / 1656 effort points; B04 (grey): Procedures on extracranial vessels; B70 (light blue): Stroke; E79 (yellow): Infections and inflammation of the respiratory organs. The final letter of the four-digit code reflects the severity of illness and resource utilization, with ‘A’ indicating the highest severity and resource use, followed by ‘B’, ‘C’, ‘D’, and ‘E’ representing progressively lower levels.

The most frequently assigned DRG was “Complex neurological treatment of acute stroke with specific operating room procedure” (*n* = 22, approximately 37% of patients), followed by “Ventilation for more than 95 hours without complex intensive care treatment” (*n* = 15, 25%) and “Craniotomy or major spinal surgery” (*n* = 8, approximately 13%). The remaining cases were categorized under DRGs reflecting intensive care and complex procedures. These included intensive care treatment for neurological conditions, prolonged mechanical ventilation with or without complex intensive care, complex craniotomy or spinal surgery, procedures involving extracranial vessels, less severe stroke presentations, and respiratory infections. The detailed distribution of billed DRGs is presented in the bar chart shown in Fig. 1.

### Health-insurance costs

The mean hospitalization cost in the control group was €22,392.89 (standard deviation €14,980.84), compared to €18,127.20 (standard deviation €7,828.65) in the PES group. The median cost was also lower in the PES group (€14,852.88) than in the control group (€18,273.38). However, these mean differences did not reach statistical significance (*p* = 0.087).

**Fig. 2:**
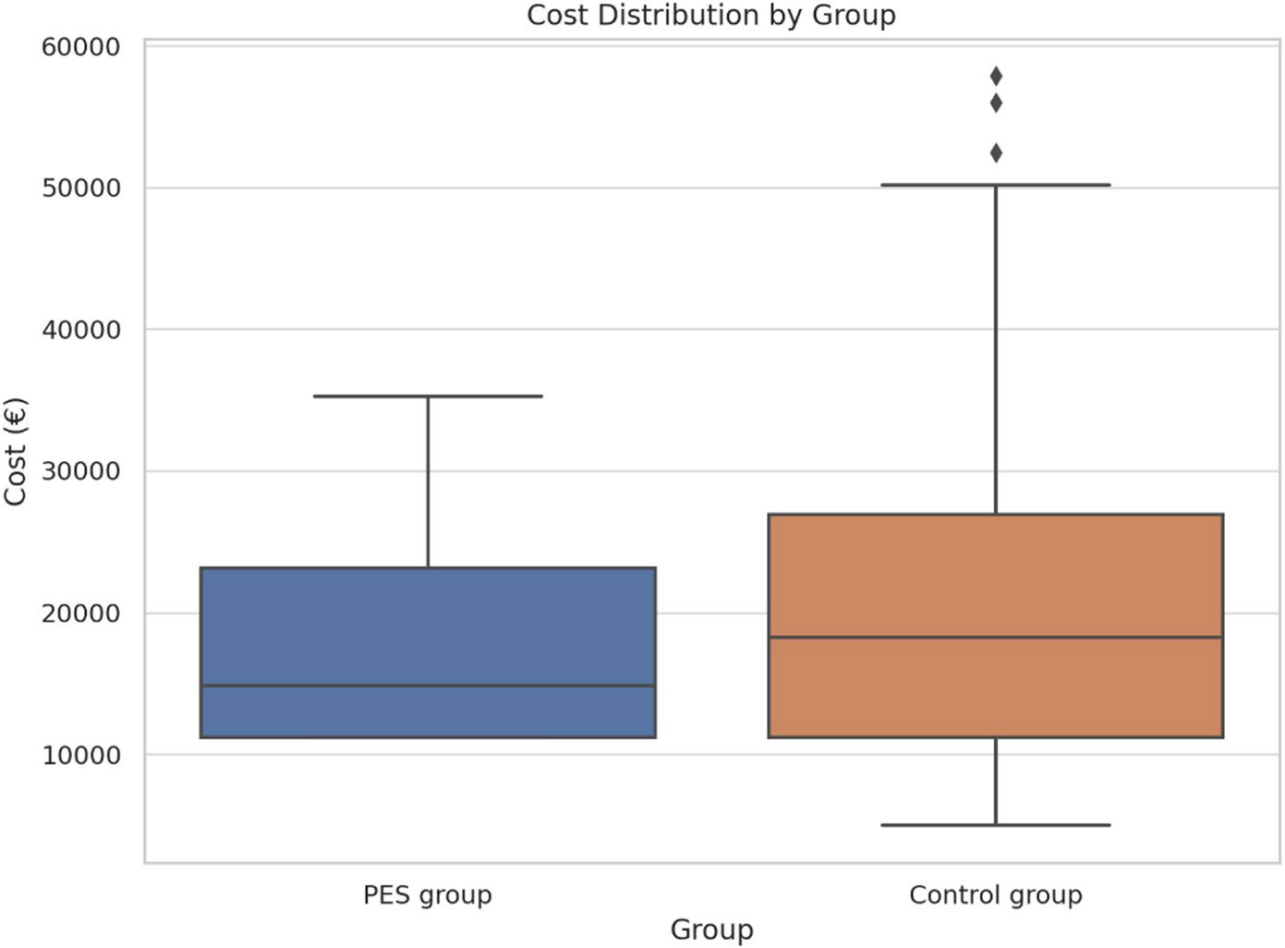
Boxplots of hospitalization costs in the intervention group (blue; Pharyngeal Electrical Stimulation, PES) and the control group (red; sham stimulation). Cost outliers were observed exclusively in the control group, where three patients incurred costs exceeding €50,593.

Notably, high-cost outliers were observed exclusively in the control group. Three patients in this group incurred hospitalization costs exceeding the cost-outlier threshold of €50,593, with the highest amount being €57,863.51. All three patients required reintubation. In these patients, mean mechanical ventilation duration prior to randomization and sham stimulation was 119.7 h, whereas mean ventilation duration following sham stimulation and subsequent reintubation was 586.7 h. In contrast, no such outliers were present in the PES group, where the maximum cost was €35,257.49. The cost distributions for both groups are illustrated in the boxplots shown in Fig. 2.

### Exploratory cost-outlier analysis

To further investigate the drivers of the exceptionally high costs in the sham group, an exploratory outlier analysis was performed. The three patients with the highest hospitalization costs in the sham group were characterized by markedly prolonged mechanical ventilation (mean 706 h vs. 81 h in the remainder of the cohort) and substantially longer hospital stays (47.7 vs. 18.8 days), whereas comorbidity levels were not higher (mean 1.0 vs. 1.93). Notably, all three outlier patients developed pneumonia, compared with 72% in the total cohort. These findings suggest that the extreme costs in these outlier cases were primarily driven by complications requiring prolonged ventilatory and ICU treatment rather than by baseline morbidity or systematic DRG case-mix differences.

## Discussion

This secondary analysis provides insights into the potential cost-saving effects of PES in stroke patients with severe postextubation dysphagia. While our findings did not demonstrate a statistically significant difference in mean hospitalization costs between the PES and control groups, the numerical trend toward lower costs in the intervention group, combined with the absence of high-cost outliers, may be clinically meaningful and warrants further investigation. These results contribute to the growing body of evidence supporting the clinical and economic relevance of addressing dysphagia in stroke patients^26–33^:

A systematic review by Marin et al. evaluated healthcare costs associated with post-stroke dysphagia and its complications, identifying moderate-quality evidence that dysphagia significantly increases healthcare expenditures despite methodological heterogeneity across studies^28^.

Muehlemann et al. analyzed administrative data from stroke cohorts in France and Switzerland and reported higher hospital costs in patients coded as dysphagic (€8,770 vs. €5,844 in France; 27,801 vs. 13,842 CHF in Switzerland), despite likely underreporting due to reliance on diagnostic codes^34^. Similarly, Bonilha et al., using U.S. Medicare claims, found that adjusted one-year costs were $4,510 higher in dysphagic patients, although prevalence based on coding was low (~ 10%)^31^.

In Taiwan, Chen et al. reported divergent findings: dysphagia was not an independent cost predictor in ischemic stroke during rehabilitation but was associated with increased hospitalization costs in hemorrhagic stroke (β = 1026)^35,36^. Wojner and Alexandrov demonstrated substantially higher acute hospital costs in tube-fed compared with non–tube-fed stroke patients in the US ($12,538 vs. $5,949), underscoring the economic impact of severe dysphagia and feeding dependency^37^.

In a clinically assessed cohort, Marin et al. observed 1.3-fold higher acute hospital costs in dysphagic versus non-dysphagic patients (€5,358 vs. €3,976), with long-term expenditures remaining elevated in dysphagic individuals up to 12 months post-stroke^27^. Likewise, Ducan et al., analyzing nationwide data from New Zealand, estimated a 12-month marginal cost attributable to dysphagia of NZD 24,200 per patient, increasing to NZD 34,000 in severe cases requiring tube feeding^33^.

A retrospective German study by Labeit et al., based on early instrumental FEES assessment and DRG reimbursement data, identified severe dysphagia (FEDSS level 6) as a major independent predictor of higher insurance costs (€25,643 vs. €7,580–€11,641 for lower severity levels), alongside prolonged ventilation and tracheotomy^30^. These results suggest that the main cost driver is not dysphagia per se but its most severe form, characterized by impaired secretion management and an increased likelihood of ICU interventions. This may help explain the absence of high-cost outliers in the PES group in the present study. PES primarily targets sensory deficits and secretion management and may have contributed to a reduction in dysphagia severity from FEDSS level 6 to a lower level. Even a one-level improvement may be sufficient to reduce the risk of high-cost outliers, for example by preventing resource-intensive complications such as reintubation, which occurred in all high-cost cases within the control group.

Finally, a systematic review by Marin et al. on cost-effectiveness of dysphagia interventions concluded that early screening and management strategies are generally cost-effective, although data on restorative therapies such as PES remain limited^26^. Our findings suggest that cost-saving benefits may extend to such interventions as well.

The literature discussed above highlights substantial methodological variability in evaluating dysphagia-related healthcare costs. First, cost analyses may adopt various perspectives, including those of hospitals, patients, insurers, or society, each of which captures different aspects of economic burden. Second, study timeframes vary widely, from acute hospitalization to long-term care. Third, dysphagia identification methods differ significantly, ranging from diagnostic codes to bedside or instrumental assessments. Fourth, cost estimation approaches also vary, including direct cost calculation, reimbursement analysis, or comparison with matched control groups. In this study, costs were analyzed from the perspective of health insurance providers. This perspective does not capture the full economic burden for hospitals or other stakeholders. Under Germany’s DRG system, reimbursement is based primarily on the principal diagnosis. Additional payments are only made under specific conditions, such as extended hospital stays. Other costly elements often linked to dysphagia, including antibiotic use or increased general nursing care, are not typically reimbursed. Therefore, actual costs incurred by hospitals likely exceed the DRG-based reimbursements analyzed here.

Another relevant consideration is the long-term impact of dysphagia on care dependency and institutionalization^38,39^. While our analysis focused on acute hospitalization costs, it is plausible that PES may yield benefits extending into the subacute and chronic phases. Longitudinal studies evaluating long-term cost trajectories are needed to fully assess the economic value of PES from a health insurance perspective.

### Limitations

This analysis has important limitations. The DRG-based reimbursement data used in this study reflect only the costs borne by the statutory health insurer during the index hospitalization. The direct costs of the PES intervention itself (including the stimulation system, disposable catheter, and procedure-related staff time) were not included. These costs currently vary by healthcare system. In Germany, they comprise a one-time investment for the base station (approximately €14,000) and per-patient usage costs for the single-use catheter (approximately €1,500). As such, the present analysis does not constitute a full cost-effectiveness evaluation, but rather an exploratory payer-perspective assessment of potential reductions in hospitalization costs. Future economic studies should incorporate the intervention costs, as well as downstream effects across care sectors, in order to determine the true net budget impact and cost-effectiveness of PES.

Further, the sample size was relatively small, which likely limited the statistical power to detect significant cost differences. The original trial was powered to assess the primary outcome of reintubation, not costs, and therefore this analysis may be underpowered. For the same reason, we did not perform multivariable regression modelling to adjust for potential confounders such as comorbidities or dysphagia severity, as the available sample size would have carried a substantial risk of statistical overfitting. Instead, we opted for an unadjusted comparison, acknowledging that the results should be interpreted as exploratory. Baseline demographic, neurological, and dysphagia-related characteristics were well balanced between groups at study inclusion; however, residual confounding cannot be fully excluded.

In addition, cost estimates were based on the German DRG reimbursement system, which reflects the perspective of statutory health insurance in Germany. As healthcare financing varies internationally, the generalizability of our findings may be limited. However, similar reimbursement systems are in use in various countries around the world. Finally, not all health insurance expenditures were captured in the DRG calculation. Nursing reimbursements (*Pflegeentgelt*) and specific additional payments (*Zusatzentgelt*, *Neue Untersuchungs- und Behandlungsmethoden*) are billed separately; however, the latter two are rarely applied in stroke care.

A further methodological consideration concerns blinding and outcome assessment. Although allocation in the parent trial was masked to outcome assessors and treating clinical staff, participants may perceive active stimulation, which could potentially influence subjective responses or behavioral performance during swallowing assessments.

Despite these limitations, the absence of high-cost outliers in the PES group is a noteworthy observation. This finding aligns with previous evidence demonstrating the clinical benefits of PES and suggests that, beyond lowering average costs, PES may help prevent extremely costly cases. These high-cost cases often have a disproportionate impact on overall stroke care expenditure.

## Conclusions

In this secondary, hypothesis-generating analysis, the difference in mean hospitalization costs between PES and sham treatment did not reach statistical significance (*p* = 0.087). However, extreme high-cost outliers were only observed in the control group and not in the PES group. This finding should be interpreted cautiously and viewed as exploratory rather than definitive. Larger, prospectively powered trials with comprehensive health-economic evaluation and extended follow-up are required to determine whether this preliminary signal translates into a reproducible reduction in high-cost cases and, ultimately, whether PES may be cost-effective. Should this be confirmed, health insurers may have a financial interest in covering the procedure, even independently of its clinical benefits.

## Data Availability

The datasets generated and/or analysed during the current study are not publicly available due to privacy and institutional restrictions but are available from the corresponding author on reasonable request.

## References

[1] Banda, K. J. et al. Prevalence of dysphagia and risk of pneumonia and mortality in acute stroke patients: a meta-analysis. *BMC Geriatr.* **22**, 420 (2022). 35562660 10.1186/s12877-022-02960-5 PMC9103417

[2] Bertschi, D. et al. Post-extubation dysphagia in the ICU-a narrative review: epidemiology, mechanisms and clinical management (Update 2025). *Crit. Care*. **29**, 244 (2025). 40524213 10.1186/s13054-025-05492-7 PMC12172361

[3] Suntrup-Krueger, S. et al. Extubation readiness in critically ill stroke patients. *Stroke* **50**, 1981–1988 (2019). 31280655 10.1161/STROKEAHA.118.024643

[4] Labeit, B. et al. The assessment of dysphagia after stroke: state of the art and future directions. *Lancet Neurol.* **22**, 858–870 (2023). 37596008 10.1016/S1474-4422(23)00153-9

[5] Martínez-Alejos, R. et al. Effects of mechanical insufflation-exsufflation on sputum volume in mechanically ventilated critically ill subjects. *Respir. Care*. **66**, 1371–1379 (2021). 34103385 10.4187/respcare.08641

[6] Stilma, W. et al. Mechanical insufflation-exsufflation for invasively ventilated critically ill patients—A focus group study. *Nurs. Crit. Care*. **28**, 923–930 (2023). 36464804 10.1111/nicc.12858

[7] Warnecke, T. et al. Neurogenic dysphagia: systematic review and proposal of a classification system. *Neurology* **96**, 876–889 (2021). 10.1212/WNL.0000000000011350 33318164

[8] Labeit, B. et al. Comparison of simultaneous swallowing endoscopy and videofluoroscopy in neurogenic dysphagia. *J. Am. Med. Dir. Assoc.*10.1016/j.jamda.2021.09.026 (2021). 34678269 10.1016/j.jamda.2021.09.026

[9] Dziewas, R. et al. Diagnosis and treatment of neurogenic dysphagia - S1 guideline of the German Society of Neurology. *Neurol. Res. Pract.* **3**, 23 (2021). 33941289 10.1186/s42466-021-00122-3 PMC8094546

[10] Dziewas, R. et al. Safety and clinical impact of FEES - results of the FEES-registry. *Neurol. Res. Pract.* **1**, 16 (2019). 33324882 10.1186/s42466-019-0021-5 PMC7650078

[11] Warnecke, T. et al. Fiberoptic endoscopic Dysphagia severity scale predicts outcome after acute stroke. *Cerebrovasc. Dis.* **28**, 283–289 (2009). 19609080 10.1159/000228711

[12] Labeit, B. et al. Dysphagia after stroke: research advances in treatment interventions. *Lancet Neurol.* **23**, 418–428 (2024). 38508837 10.1016/S1474-4422(24)00053-X

[13] Dziewas, R. et al. European stroke organisation and european society for swallowing disorders guideline for the diagnosis and treatment of post-stroke dysphagia. *Eur Stroke J* **6**, (2021). 10.1177/23969873211039721 PMC8564153 34746431

[14] Hamdy, S., Aziz, Q., Rothwell, J. C., Hobson, A. & Thompson, D. G. Sensorimotor modulation of human cortical swallowing pathways. *J. Physiol.* **506** (Pt 3), 857–866 (1998). 9503343 10.1111/j.1469-7793.1998.857bv.x PMC2230741

[15] Muhle, P. et al. Targeting the sensory feedback within the swallowing network-Reversing artificially induced pharyngolaryngeal hypesthesia by central and peripheral stimulation strategies. *Hum. Brain Mapp.*10.1002/hbm.25233 (2020). 33068056 10.1002/hbm.25233 PMC7776007

[16] Suntrup, S. et al. Pharyngeal electrical stimulation can modulate swallowing in cortical processing and behavior - magnetoencephalographic evidence. *Neuroimage* **104**, 117–124 (2015). 25451471 10.1016/j.neuroimage.2014.10.016

[17] Suntrup, S. et al. Electrical pharyngeal stimulation for dysphagia treatment in tracheotomized stroke patients: a randomized controlled trial. *Intensive Care Med.* **41**, 1629–1637 (2015). 26077087 10.1007/s00134-015-3897-8

[18] Dziewas, R. et al. Pharyngeal electrical stimulation for early decannulation in tracheotomised patients with neurogenic dysphagia after stroke (PHAST-TRAC): a prospective, single-blinded, randomised trial. *Lancet Neurol.* **17**, 849–859 (2018). 30170898 10.1016/S1474-4422(18)30255-2

[19] Bath, P. M. et al. Pharyngeal electrical stimulation for neurogenic dysphagia following stroke, traumatic brain injury or other causes: Main results from the PHADER cohort study. *EClinicalMedicine* **28**, 100608 (2020). 33294818 10.1016/j.eclinm.2020.100608 PMC7700977

[20] Chiang, C. F. et al. Comparative efficacy of noninvasive neurostimulation therapies for acute and subacute poststroke dysphagia: a systematic review and network meta-analysis. *Arch. Phys. Med. Rehabil*. **100**, 739–7504 (2019). 30352222 10.1016/j.apmr.2018.09.117

[21] Wang, T. et al. Comparative efficacy of non-invasive neurostimulation therapies for poststroke dysphagia: A systematic review and meta-analysis. *Neurophysiol. Clin.* **51**, 493–506 (2021). 34535361 10.1016/j.neucli.2021.02.006

[22] Cheng, I., Sasegbon, A. & Hamdy, S. Effects of neurostimulation on poststroke dysphagia: a synthesis of current evidence from randomized controlled trials. *Neuromodulation* **24**, 1388–1401 (2021). 33301231 10.1111/ner.13327 PMC9292042

[23] Suntrup-Krueger, S. et al. Treating postextubation dysphagia after stroke with pharyngeal electrical stimulation -insights from a randomized controlled pilot trial. *Neurotherapeutics*10.1016/j.neurot.2025.e00613 (2025). 40383663 10.1016/j.neurot.2025.e00613 PMC12418440

[24] Suntrup-Krueger, S. et al. Pharyngeal electrical stimulation for postextubation dysphagia in acute stroke: a randomized controlled pilot trial. *Crit. Care*. **27**, 383 (2023). 37789340 10.1186/s13054-023-04665-6 PMC10548555

[25] Institut für das Entgeltsystem im Krankenhaus GmbH (InEK). *German Diagnosis Related Groups, aG-DRG-Version 2025 Definitionshandbuch Kompaktversion* (Siegburg, 2024).

[26] Marin, S. et al. Economic evaluation of clinical, nutritional and rehabilitation interventions on oropharyngeal dysphagia after stroke: A Systematic review. *Nutrients* 15, (2023). 10.3390/nu15071714 PMC10097035 37049553

[27] Marin, S. et al. Healthcare costs of post-stroke oropharyngeal dysphagia and its complications: malnutrition and respiratory infections. *Eur. J. Neurol.* **28**, 3670–3681 (2021). 34176195 10.1111/ene.14998

[28] Marin, S., Serra-Prat, M., Ortega, O. & Clavé, P. Healthcare-related cost of oropharyngeal dysphagia and its complications pneumonia and malnutrition after stroke: a systematic review. *BMJ Open.* **10**, 031629 (2020). 10.1136/bmjopen-2019-031629 PMC7418658 32784251

[29] Attrill, S., White, S., Murray, J., Hammond, S. & Doeltgen, S. Impact of oropharyngeal dysphagia on healthcare cost and length of stay in hospital: a systematic review. *BMC Health Serv. Res.* **18**, 594 (2018). 30068326 10.1186/s12913-018-3376-3 PMC6090960

[30] Labeit, B. et al. Costs of post-stroke dysphagia during acute hospitalization from a health-insurance perspective. *Eur. Stroke J.* **23969873221147740**10.1177/23969873221147740 (2022). 10.1177/23969873221147740 PMC10069210 37021194

[31] Bonilha, H. S. et al. The one-year attributable cost of post-stroke dysphagia. *Dysphagia* **29**, 545–552 (2014). 24948438 10.1007/s00455-014-9543-8 PMC4179977

[32] Qureshi, A. I. et al. Annual direct cost of dysphagia associated with acute ischemic stroke in the United States. *J. Stroke Cerebrovasc. Dis.* **31**, 106407 (2022). 35259613 10.1016/j.jstrokecerebrovasdis.2022.106407

[33] Duncan, S., Menclova, A., Huckabee, M. L., Cadilhac, D. A. & Ranta, A. How much does dysphagia cost? understanding the additional costs of dysphagia for new zealand in patients hospitalised with stroke. *Neuroepidemiology* **59**, 57–67 (2025). 38718760 10.1159/000539133

[34] Muehlemann, N. et al. Hospital costs impact of post ischemic stroke dysphagia: Database analyses of hospital discharges in France and Switzerland. *PLoS One*. **14**, 0210313 (2019). 10.1371/journal.pone.0210313 PMC6328123 30629665

[35] Chen, C. M., Chang, C. H., Hsu, H. C., Lin, C. H. & Chen, K. H. Factors predicting the total medical costs associated with first-ever ischeamic stroke patients transferred to the rehabilitation ward. *J. Rehabil Med.* **47**, 120–125 (2015). 25268933 10.2340/16501977-1894

[36] Chen, C. M. & Ke, Y. L. Predictors for total medical costs for acute hemorrhagic stroke patients transferred to the rehabilitation ward at a regional hospital in Taiwan. *Top. Stroke Rehabil*. **23**, 59–66 (2016). 26094779 10.1179/1945511915Y.0000000006

[37] Wojner, A. W. & Alexandrov, A. V. Predictors of tube feeding in acute stroke patients with dysphagia. *AACN Clin. Issues*. **11**, 531–540 (2000). 11288417 10.1097/00044067-200011000-00006

[38] Smithard, D. G., Smeeton, N. C. & Wolfe, C. D. A. Long-term outcome after stroke: does dysphagia matter? *Age Ageing*. **36**, 90–94 (2007). 17172601 10.1093/ageing/afl149

[39] Arnold, M. et al. Dysphagia in acute stroke: incidence, burden and impact on clinical outcome. *PLoS ONE*. **11**, e0148424 (2016). 26863627 10.1371/journal.pone.0148424 PMC4749248

